# Effects of Thai propolis mixed in mineral trioxide aggregate on matrix metalloproteinase-2 expression and activity in inflamed human dental pulp cells

**DOI:** 10.1590/1678-7757-2024-0168

**Published:** 2024-09-20

**Authors:** Nutnicha TIYAPITSANUPAISAN, Nutthapong KANTRONG, Subin PUASIRI, Anupong MAKEUDOM, Suttichai KRISANAPRAKORNKIT, Pattama CHAILERTVANITKUL

**Affiliations:** 1 Khon Kaen University Faculty of Dentistry Department of Restorative Dentistry Khon Kaen Thailand Khon Kaen University, Faculty of Dentistry, Department of Restorative Dentistry, Khon Kaen 40002, Thailand.; 2 Mae Fah Luang University School of Dentistry Chiang Rai Thailand Mae Fah Luang University, School of Dentistry, Chiang Rai 57100, Thailand.; 3 Khon Kaen University Faculty of Dentistry Department of Preventive Dentistry Khon Kaen Thailand Khon Kaen University, Faculty of Dentistry, Department of Preventive Dentistry, Khon Kaen 40002, Thailand.

**Keywords:** Dental pulp, Matrix metalloproteinase-2, Mineral trioxide aggregate, Propolis

## Abstract

**Objectives:**

This study sought to determine effects of Thai propolis extract mixed in mineral trioxide aggregate (MTA) on matrix metalloproteinase-2 (MMP-2) expression and its activity in inflamed human dental pulp cells (HDPCs).

**Materials and Methods:**

Interleukin-1β-primed HDPCs were treated with either the eluate of MTA mixed with distilled water, of MTA mixed with 0.75 mg/ml of the propolis extract, or of Dycal^®^, 0.75 mg/ml of the propolis extract, or 0.2% (v/v) of chlorhexidine for 24 or 72 h. The viability of HDPCs was determined by the PrestoBlue^®^ cytotoxic assay. HDPCs’ lysates were analyzed for MMP-2 mRNA expression by RT-qPCR, while their supernatants were measured for MMP-2 activity by gelatin zymography.

**Results:**

At 24 and 72 h, a non-toxic dose of the propolis extract at 0.75 mg/ml by itself or mixed in MTA tended to reduce MMP-2 expression upregulated by MTA, while it further decreased the MMP-2 activity as compared to that of MTA mixed with distilled water. The MMP-2 activity of interleukin-1β-primed HDPCs treated with the eluate of the propolis extract mixed in MTA was significantly lower than that of interleukin-1β-primed HDPCs at 24 h (p=0.012). As a control, treatment with chlorhexidine significantly inhibited MMP-2 expression induced by MTA and MMP-2 activity enhanced by interleukin-1β (p<0.05). Treatment with Dycal^®^ caused a significant increase in HDPC’s death, resulting in a significant decrease in MMP-2 expression and activity (p<0.05).

**Conclusions:**

MTA mixed with Thai propolis extract can reduce MMP-2 mRNA expression and activity when compared to MTA mixed with distilled water in inflamed HDPCs.

## Graphical abstract

**Figure:**
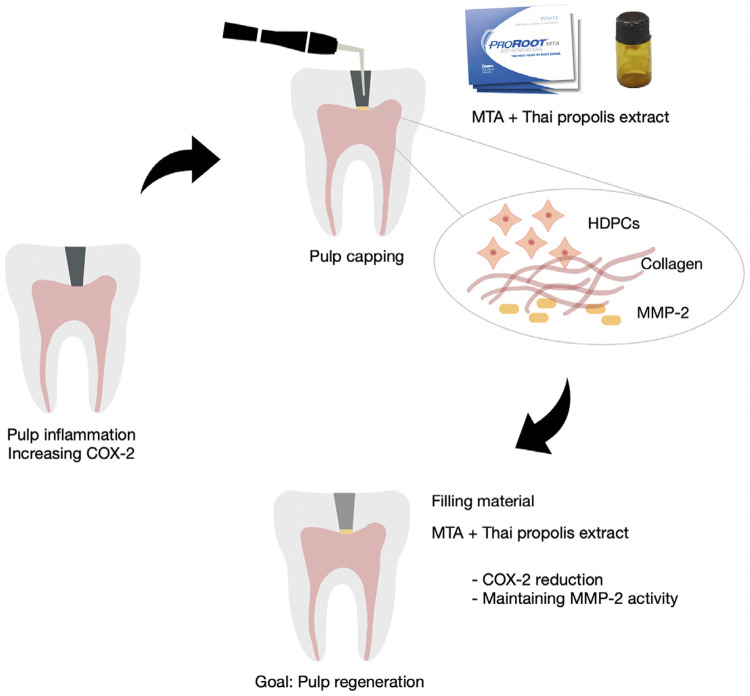

## Introduction

Extracellular matrices (ECMs) are an integral part of development, morphogenesis, and tissue remodeling of several organs. The integrity of ECMs is affected by matrix metalloproteinase (MMP), which plays an essential role in tissue remodeling.^1^ Among the MMP family, gelatinases, including MMP-2 and MMP-9, have a crucial role in soft tissue and dentine collagen breakdown.^2,3^ Such molecular events are prerequisites for pulpal repair mechanism in response to deep dental caries.^2^ Both MMPs are synthesized and released by human dental pulp cells (HDPCs) and osteoblasts.^1,4^ It has been shown that the pro-inflammatory interleukin (IL)-1β, ubiquitously expressed in caries-infected dental pulp,^5^ can induce MMP-2 levels in HDPCs.^6^ Moreover, in comparison with normal pulp, MMP-2 expression was upregulated in acute pulpitis,^7^ suggesting that MMP-2 plays a pivotal role in pulp inflammation. There is also a relationship between MMP-2 and cyclooxygenase-2 (COX-2)/prostaglandin E2 (PGE2) in HDPCs, since the production of MMP-2 is shown to be suppressed by a specific inhibitor of COX-2.^8^

Vital pulp therapy recruits HDPCs to produce ECM that will later turn to be a scaffold for the mineralized tissue barrier across the exposed surface, commonly referred to dentine bridge.^9^ Pulp capping materials are one of the essential factors that contribute to the success of vital pulp therapy.^10^ Calcium hydroxide has been a material of choice for pulp capping for decades due to its healing potential by production of dentine bridge.^11^ However, this bridge can be degraded over time, creating tunnel defects in the newly formed dentine that leads to unpredictable outcomes for pulp capping with calcium hydroxide.^11^ This disadvantage has prompted clinicians to turn towards bioceramic materials such as mineral trioxide aggregate (MTA) or biodentine. MTA is the first bioceramic material with biocompatibility and antibacterial properties.^11,12^ It maintains the integrity of pulp tissue better than calcium hydroxide.^11-13^ Despite these advantages of MTA, its unfavorable properties include delayed setting time, tooth staining over time, difficult handling, and high costs.^14,15^

Propolis or bee glue possesses antimicrobial and anti-inflammatory properties.^16,17^Flavonoids, one of its major active components, can inhibit expression of IL-1 via inactivation of nuclear factor-kappa B (NF-kB) in dental pulp.^17^ Thai propolis extract suppresses pro-inflammatory COX-2 expression and PGE2 levels via NF-kB in inflamed HDPCs.^18^ The promotion of Thai propolis extract for pulp wound healing in pulp-exposed rabbits’ teeth is evidenced by less inflammation and more well-organized dentinal tubules within the dentine bridge than those treated with calcium hydroxide.^19^ Moreover, propolis has demonstrated better anti-inflammatory activity against MMP-2 and MMP-9 than Biodentine in tooth stem cells.^20^ Whereas propolis suppresses IL-1 expression more than MTA, MTA instead promotes mineralization better than propolis in HDPCs.^21^ Therefore, a combination of propolis and MTA may be beneficial for clinical use as a pulp capping material to reduce inflammation, while simultaneously inducing mineralization. Two recent studies have shown that the combination of propolis and calcium hydroxide results in diminished activation of inflammation, while producing more type I collagen than calcium hydroxide only.^22,23^ It is, therefore, relevant to determine whether the combination of MTA and Thai propolis extract, which has not yet been studied, can modify an inflammatory process during the initial stage of pulp wound healing. Considering the anti-inflammatory properties of propolis, our extended hypothesis was that propolis used as vehicle for MTA preparation might enhance the pulpal repair potential of dental pulp cells. The objective of this study was to investigate the effects of Thai propolis extract mixed in MTA on MMP-2 expression and activity in inflamed HDPCs.

## Methodology

The extraction of Thai propolis was modified from our original protocol.^19^ Briefly, propolis, collected from an apiary in the Nong Khai province, Thailand, was ground into fine particles and dissolved in 70% (v/v) of analytical ethanol. The mixture was vortexed, followed by ultrasonic-assisted extraction at 60 Hz for 20 min at room temperature. The mixture was centrifuged at 9,167 g for 5 min, and the supernatant was collected in a new tube. The extraction was repeated three times, and the supernatant was combined, dried overnight with a rotary evaporator, and redissolved in absolute ethanol with the stock concentration at 18.3 mg/ml and the yield of extraction at 2.83% (w/w). The extract was kept in darkness and stored at −20 °C until further use.

### Culture of primary human dental pulp cells

**Figure 1:**
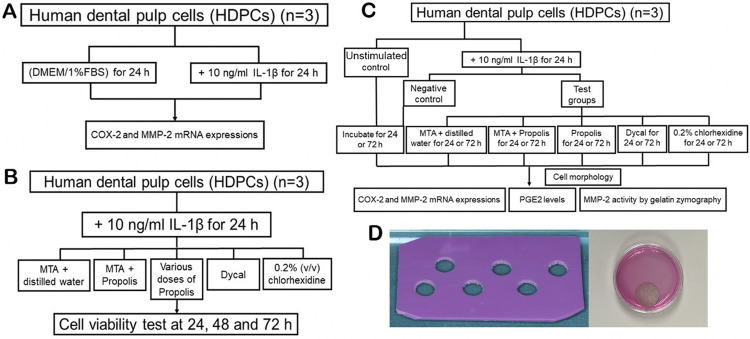
*In vitro* experimental designs of this study. Three flow charts (A, B, C) illustrating each research design of this study. Each experiment was repeated three times for each of the three different human dental pulp cells (HDPCs), isolated from three different donors (n = 3). D) The picture shows a vinylpolysiloxane-added silicone mold and the prepared MTA mixed with Thai propolis extract at 0.75 mg/ml which was immersed in DMEM for 24 h.

Normal pulp tissues were obtained from three freshly extracted upper or lower nonfunctional third molars from three healthy volunteers (2 females and 1 male; 18-25 years old) with their informed consent. These teeth exhibited no evidence of carious lesions, cracks, restorations, or periodontal diseases. The research protocol was approved by the Khon Kaen University Ethics Committee in Human Research (HE6662052). By simple exodontia, the pulp within each tooth was removed by separating the tooth with a carborundum disc and extraction forceps. The removed tissue was immediately submerged in a 35-mm culture dish, containing Dulbecco’s Modified Eagle Medium (DMEM). HDPCs, overgrown from the explants, were sub-cultured for expansion of their cell numbers after they reached 80% cell confluence.^18^ The experiments in this study comprised: i) determination of MMP-2 and COX-2 mRNA induction by stimulation with IL-1β at 10 ng/ml for 24 h in HDPCs (Figure 1A), ii) a cell viability test upon treatment with various agents for 24, 48, or 72 h in HDPCs stimulated with IL-1β for 24 h (Figure 1B), and iii) a study of MMP-2 expression/activity and COX-2 expression/PGE2 levels in the primed HDPCs treated with eluates from different agents for 24 or 72 h (Figure 1C). Each experiment was performed in triplicate separately for each of the three cell lines, derived from three different donors.

### Preparation of material eluates

White ProRoot^®^ MTA and Dycal^®^ (Dentsply International Inc., DE, USA) were prepared according to the manufacturers’ instructions at the dimensions of 10 mm in diameter and 1 mm in thickness in a vinylpolysiloxane-added silicone mold (Figure 1D). The ProRoot^®^ MTA was mixed with distilled water or the highest non-toxic concentration of the Thai propolis extract, determined by the PrestoBlue^®^ cytotoxic assay, at aratio of 3:1, and the mixture was left until setting for 24 h at 37 °C in 100% humidity before removal from the mold.^21^ All materials, including MTA mixed with distilled water, MTA mixed with the propolis extract (Figure 1D), and Dycal^®^, were immersed in 5 ml of 1% FBS-containing DMEM for 24 h according to ISO 10993-5:2012.^24^ The MTA discs were sterilized by ultraviolet irradiation for 30 min^25^ before use to elute their contents, then filtered through 0.22-μm pore size membrane, and kept at -20°C until being used.^21^

### Cytotoxicity of Thai propolis extract and material eluates

The cells at 1×10^4^ concentration were seeded into each well of a 96-well plate (Corning Incorporated, NY, USA) and cultured in DMEM with 1% FBS at 37°C for 24 h. On the following day, the cells were stimulated with 100 μl human recombinant IL-1β (R&D Systems, Inc., Minneapolis, MN, USA) at 10 ng/ml for 24 h to simulate the state of pulp cell inflammation.^18^ The primed cells were treated with 100 μl of 0.25-1.50 mg/ml Thai propolis extract, 100 μl of the eluate released from MTA mixed with either distilled water, the highest non-toxic concentration of the propolis extract, or released from Dycal^®^, or at a concentration of 0.2% (v/v) of chlorhexidine for 24, 48, or 72 h (Figure 1B). Cells with no treatment or primed cells treated with 10% (v/v) of dimethyl sulfoxide (DMSO) served as a negative or a positive control for the cytotoxic assay, respectively. After treatment, the culture supernatants were removed, and the cells were incubated with a 50 μl volume of the PrestoBlue^®^ reagent per well for 60 min. The fluorescence activity was measured at 560/590 nm using a microplate reader (Varioskan Flash, Thermo Fisher Science, Vanntaa, Finland). The percentage of cell viability was computed as previously described.^18^ The highest non-toxic concentration of the propolis extract was selected to mix with ProRoot^®^ MTA at the aforementioned ratio.

### Cell treatment

After being treated with 10 ng/ml IL-1β for 24 h, the IL-1β-stimulated HDPCs, seeded at 3×10^4^ cells per well in a 6-well plate, were divided into four experimental groups, including: 1) treatment with a 1.5 ml volume of the eluate released from ProRoot^®^ MTA mixed with distilled water, 2) ProRoot^®^ MTA mixed with the highest non-toxic concentration of the propolis extract, 3) with Dycal^®^, and 4) 1.5 ml of 1% FBS-containing DMEM that contained the highest non-toxic concentration of the propolis extract (Figure 1C). None of the eluates used to treat IL-1β-primed HDPCs were diluted, since the undiluted eluate released from MTA has not been found to be toxic to HDPCs.^26,27^ A positive control group was the treatment with 1.5 ml of 1% FBS-containing DMEM, at 0.2% concentration (v/v) of chlorhexidine, while a negative control group was IL-1β-stimulated HDPCs, cultured in 1% FBS-containing DMEM without any treatment. We used chlorhexidine in this study due to its direct inhibition against the expression of MMP-2,^28^ our target gene of interest. The unstimulated control group was HDPCs, cultured in 1% FBS-containing DMEM, which were neither stimulated nor treated. The cells were harvested for expressions of MMP-2 and COX-2 mRNA, whereas the conditioned media were collected for determination of MMP-2 activity and PGE2 levels.

### RNA isolation and reverse transcription-quantitative polymerase chain reaction (RT-qPCR)

Total RNA was extracted using the RNA and protein purification kit (NucleoSpin^®^ RNA/Protein, Dueren, Germany) according to the manufacturer’s protocol. An RT-qPCR analysis was performed by a two-step procedure, in which complementary DNA (cDNA) was synthesized from a 500 ng amount of total RNA using the High-Capacity cDNA Reverse Transcription kit (Thermo Fisher Scientific). A 20 μl volume of qPCR included 5% (v/v) of cDNA, 10 μl of the SYBR Green master mix (PowerUp SYBR Green Master Mix, Applied Biosystems by Thermo Fisher Scientific), and 10 μM of each pair of oligonucleotide primers specific for COX-2,^18^ MMP-2,^29^ or glyceraldehyde 3-phosphate dehydrogenase (GAPDH),^18^ as a housekeeping gene control. PCR consisted of denaturation at 95 °C for 20 s, annealing at 60°C for 20 s, and extension at 72 °C for 10 s, for 40 cycles, using the LightCycler^®^ 480 instrument (Roche Molecular Biochemicals, Mannheim, Germany). By the ΔΔCt method, a fold change in MMP-2 or COX-2 mRNA expression, normalized by GAPDH mRNA expression, in each experimental sample relative to that in the unstimulated control, set to 1.0, was computed.

### Gelatin zymography

The conditioned media, collected from samples and controls, were mixed with 5X non-reducing sample buffer at a ratio of 6:1, and a 20 μl aliquot was electrophoresed on 10% polyacrylamide gel, copolymerized with 3% (w/v) of gelatin. After electrophoresis, sodium dodecyl sulfate was removed from the gel, and the gel was rinsed twice with 2.5% (v/v) of Triton X-100 for 30 min at room temperature and incubated with 0.05 M Tris (pH 7.5), 5 mM CaCl_2_, and 1 μM ZnCl_2_ at 37 °C overnight. The gel was stained with 0.25% (w/v) of Coomassie brilliant blue solution and de-stained with 54% (v/v) of methanol and 8% (v/v) of acetic acid. The gel was scanned using an Epson scanner and converted into a digital image. The densitometry of clear bands from gelatin digestion, which indicates the activity of pro-MMP-2 and active MMP-2, was performed using ImageJ software version 1.41 (National Institutes of Health, Bethesda, MD, USA).^30,31^ Changes in the activities of combined pro-MMP-2 and active MMP-2 of experimental samples, when compared to the unstimulated control, were reported as the percentage values.

### PGE2 ELISA

The conditioned media, collected from the samples and controls, for the levels of secreted PGE2 by a competitive ELISA, as previously described^18^ using a pre-coated PGE2 quantitation kit (KGE004B; Prostaglandin E2 Parameter Assay Kit, R&D Systems, Inc). The level of secreted PGE2 in each sample was derived from a comparison with the average absorbance of known PGE2 concentrations.

### Statistical analysis

All data are presented as mean±standard deviation. Differences in mean percentages of cell viability were tested by one-way ANOVA and Bonferroni’s post-hoc comparison. Differences in average folds of COX-2 and MMP-2 mRNA expressions and in mean percentages of changes in the MMP-2 activity were tested by Kruskal-Wallis and Dunn’s Bonferroni’s post hoc test. Differences in PGE2 levels were tested by one-way ANOVA and Dunnett’s post-hoc test. Using SPSS^®^ software version 27.0 for Windows (IBM, Chicago, IL, USA), differences between samples and controls were considered statistically significant at the *p*-value<0.05.

## Results

### Cytotoxicity of Thai propolis extract, MTA, Dycal® and chlorhexidine

**Figure 2:**
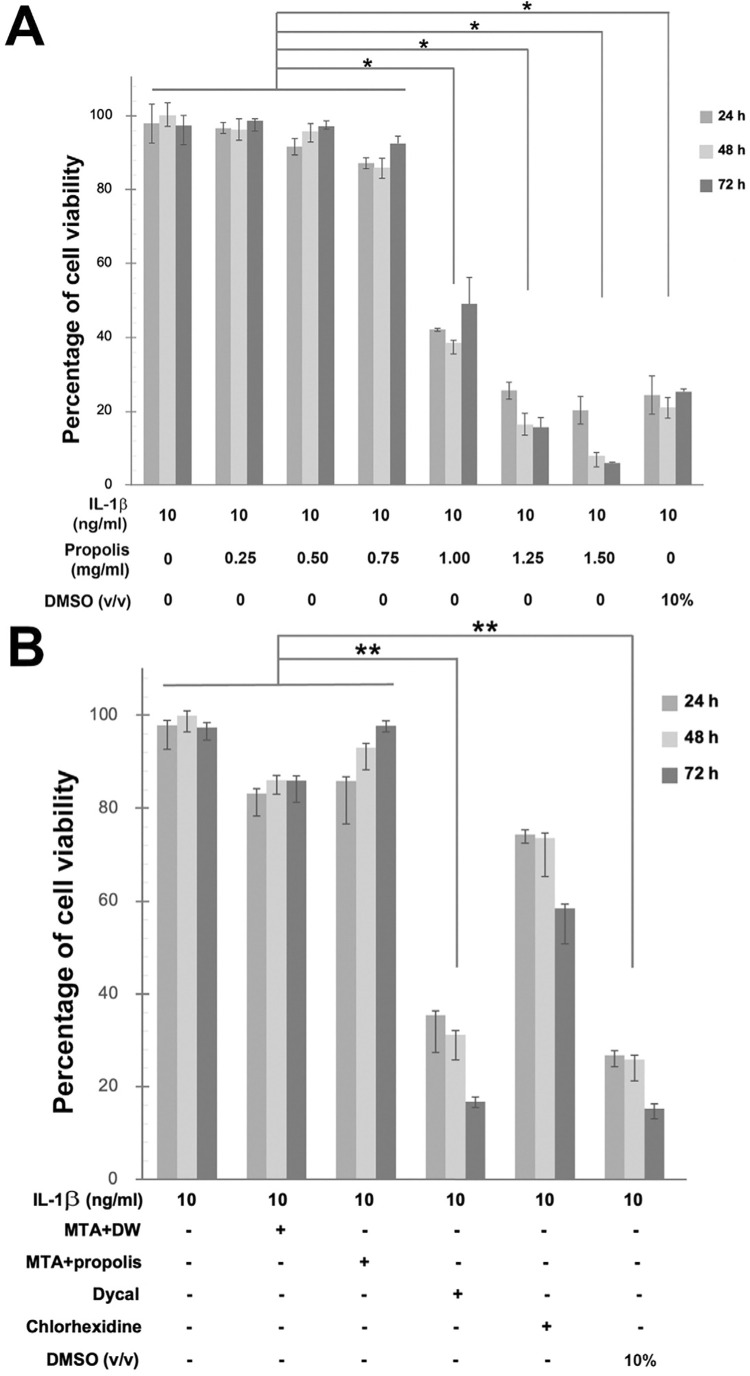
HDPCs viability test by PrestoBlue assay. Bar graphs demonstrating the percentages of cell viability upon treatment of IL-1β-primed HDPCs for 24, 48, or 72 h with indicated doses of Thai propolis extract (A) or with 100 ml of the eluate released from MTA mixed with distilled water (DW), that from MTA mixed with Thai propolis extract at 0.75 mg/ml, or that from Dycal®, or 100 ml of 0.2% (v/v) of chlorhexidine (B). Treatment for 24, 48, or 72 h with 10% (v/v) of dimethyl sulfoxide (DMSO) in A and B was used as positive control. Thai propolis extract at 0.75 mg/ml was demonstrated to be the highest, most non-toxic dose used for the subsequent experiments. Error bars = standard deviation; ** = *p*<0.01; * = *p*<0.05.

The cytotoxicity of various doses of Thai propolis extract in IL-1β-primed HDPCs was determined. It was demonstrated that the concentrations of the extract at 1 mg/ml or higher significantly decreased the percentages of cell viability at all three treatment periods, including 24, 48, and 72 h, when compared to the doses of the extract at 0.75 mg/ml or lower or no treatment control (*p*<0.05; Figure 2A). Therefore, the highest non-toxic concentration of Thai propolis extract chosen for subsequent experiments was 0.75 mg/ml. In addition, treatment of IL-1β-primed HDPCs with the eluate of MTA mixed with distilled water or mixed with the propolis extract at 0.75 mg/ml, or with 0.2% (v/v) of chlorhexidine did not significantly diminish the percentages of cell viability at all three treatment periods whereas treatment with the eluate of Dycal^®^ significantly decreased the percentages of cell viability (*p*<0.05; Figure 2B). Treatment of IL-1β-primed HDPCs with 10% (v/v) of DMSO, as a positive control for cell death, significantly reduced the percentages of cell viability at all three treatment periods (*p*<0.05; Figure 2A and B).

**Figure 3:**
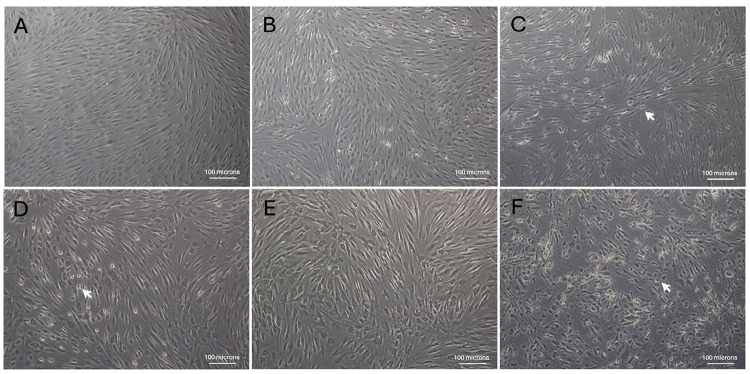
Microscopic imaging of HDPCs. Representative images showing cultured HDPCs upon treatment for 24 h (A, B, C) or 72 h (D, E, F) with 1.5 ml of the eluate released from MTA mixed with distilled water (A, D), from MTA mixed with Thai propolis extract at 0.75 mg/ml (B, E), or from Dycal® (C, F). White arrows in C, D, and F indicate rounded-shape dead cells in the culture.

The toxicity of the eluate released from Dycal^®^ in IL-1β-primed HDPCs was morphologically verified by loss of a spindle cell shape, a characteristic of mesenchymal cells, and an increased number of rounded-shape cells, indicating dead cells in the culture, at 24 h (a white arrow; Figure 3C) and 72 h (a white arrow; Figure 3F). Notably, treatment with the eluate of MTA mixed with distilled water for 72 h also caused an increase in the number of rounded-shape cells (a white arrow; Figure 3D), in contrast with none observed at 24 h (Figure 3A) or none by incubation with the eluate of MTA mixed with Thai propolis extract at 0.75 mg/ml for 72 h (Figure 3E). Although there appeared to be some dying IL-1β-primed HDPCs treated with MTA mixed with distilled water at 24 h, attached HDPCs observed under microscope remained confluent and displayed normal healthy morphological characteristics, which is consistent with a quantitation of proliferating, live cells using the PrestoBlue^®^ assay.

### Effects of Thai propolis extract mixed in MTA on MMP-2 mRNA expression and activity

**Figure 4:**
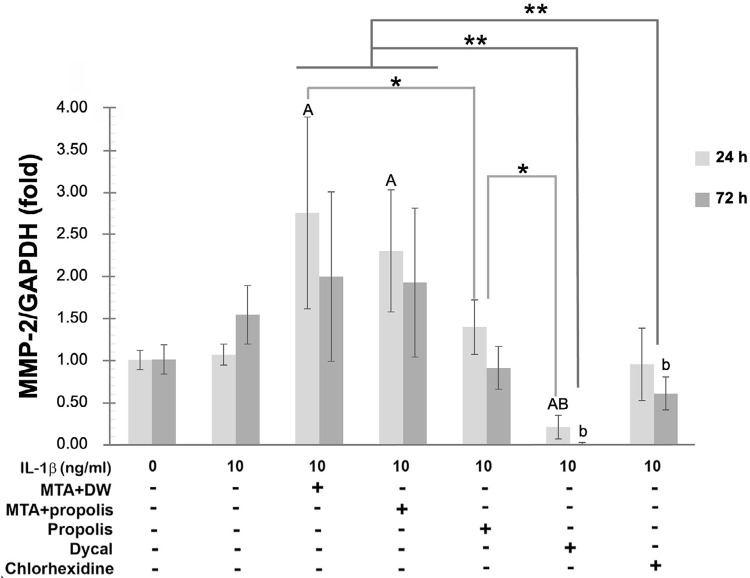
A potential reduction of MMP-2 transcripts in MTA-treated HDPCs by propolis. A bar graph demonstrating folds of MMP-2 mRNA expression, normalized by GAPDH mRNA expression, in IL-1β-primed HDPCs treated for 24 h (light gray) or 72 h (dark gray) with 1.5 ml of the eluate released from MTA mixed with distilled water (DW), from MTA mixed with Thai propolis extract at 0.75 mg/ml, or from Dycal®, Thai propolis extract at 0.75 mg/ml, or 0.2% (v/v) of chlorhexidine relative to normalized MMP-2 mRNA expression of HDPCs that were neither stimulated nor treated, set to 1.0. Error bars = standard deviation; * = *p*<0.05 for comparisons at 24 h; ** = *p*<0.001 for comparisons at both 24 and 72 h. A = *p*<0.05, compared to unstimulated HDPCs at 24 h; B = *p*<0.05, compared to IL-1β-stimulated HDPCs that were not treated for 24 h; b = *p*<0.05, compared to IL-1β-stimulated HDPCs that were not treated for 72 h.

Although stimulation with IL-1β at 10 ng/ml for 24 or 72 h failed to significantly upregulate MMP-2 mRNA, treatment of IL-1β-primed HDPCs with the eluate of MTA mixed with distilled water or with Thai propolis extract at 0.75 mg/ml significantly induced MMP-2 mRNA expression at 24 h (*p*<0.05; Figure 4). The propolis extract either used by itself or mixed in MTA tended to decrease MMP-2 mRNA expression upregulated by MTA at 24 and 72 h with the significant reduction observed by treatment with the propolis extract by itself at 24 h (*p*<0.05; Figure 4). Treatment with 0.2% (v/v) of chlorhexidine significantly decreased MMP-2 expression as compared to that with the eluate of MTA at 24 and 72 h (*p*<0.05; Figure 4).

**Figure 5:**
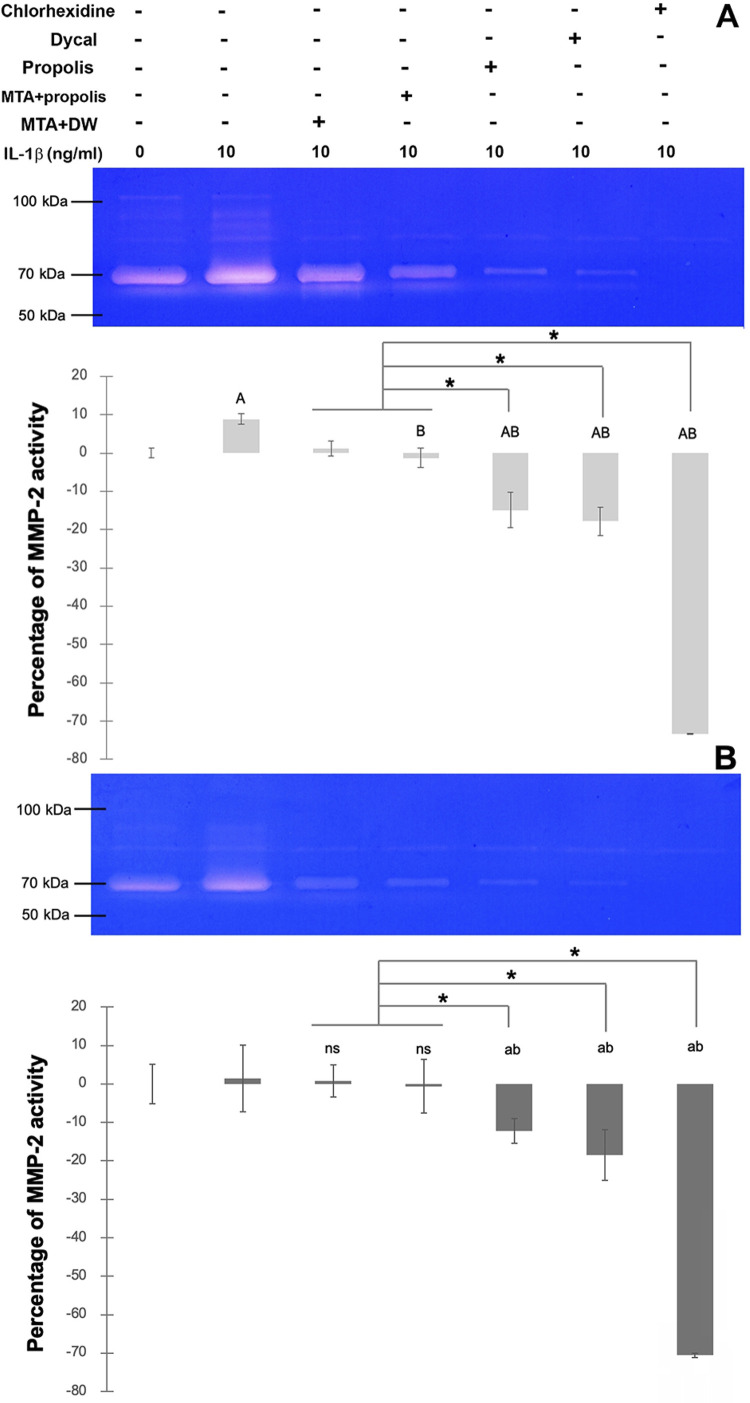
Gelatin Zymography for MMP-2 detection. Representative gelatin zymograms from the conditioned media of unstimulated HDPCS and that of IL-1β-primed HDPCs treated for 24 h (A) or 72 h (B) with 1.5 ml of the eluate released from MTA mixed with distilled water (DW), from MTA mixed with Thai propolis extract at 0.75 mg/ml, or from Dycal®, Thai propolis extract at 0.75 mg/ml, or 0.2% (v/v) of chlorhexidine. MTA mixed with either distilled water or Thai propolis extract dampened the degree of MMP-2 expression. Bar graphs below the zymograms illustrate changes in the percentages of MMP-2 activity, as measured by the densitometry of combined clear zones between pro-MMP-2 and active MMP-2, in the samples relative to that in the unstimulated HDPCs. Error bars = standard deviation; * = *p*<0.05. A = *p*<0.05, compared to unstimulated HDPCs at 24 h; B = *p* < 0.05, compared to IL-1β-stimulated HDPCs that were not treated for 24 h; a = *p* < 0.05, compared to unstimulated HDPCs at 72 h; b = *p*<0.05, compared to IL-1β-stimulated HDPCs that were not treated for 72 h, ns = non-significant, compared to IL-1β-stimulated HDPCs that were not treated for 72 h.

Despite the inability of IL-1β to induce MMP-2 mRNA expression at 24 or 72 h as aforementioned, the combined activity of pro-MMP-2 and active MMP-2 was found to be significantly increased by treatment with IL-1β at 24 h (*p*<0.05; Figure 5A), but not at 72 h (Figure 5B), when compared to the unstimulated control. In contrast to the inducible effect of the eluate of MTA on MMP-2 mRNA expression (Figure 5), treatment with the eluate of MTA mixed with either distilled water or the propolis extract or with the propolis extract by itself evidently diminished the combined MMP-2 activity with the significant reduction observed only by treatment with the propolis extract by itself at 24 and 72 h (*p*<0.05; Figure 5A and B). As a positive control for inhibition of the combined MMP-2 activity in the conditioned media,^33^treatment with 0.2% (v/v) of chlorhexidine completely abolished the combined MMP-2 activity in inflamed HDPCs at 24 and 72 h (*p*<0.05; Figure 5A and B). Providing that an equal volume of HDPCs culture supernatants was assayed, significant decreases in the MMP-2 mRNA expression (*p*<0.05; Figure 4) and in the MMP-2 activity (*p*<0.05; Figure 5) by treatment with the eluate of Dycal^®^ at 24 and 72 h was observed.

### Effects of Thai propolis extract mixed in MTA on COX-2 mRNA expression and PGE2 levels

**Figure 6:**
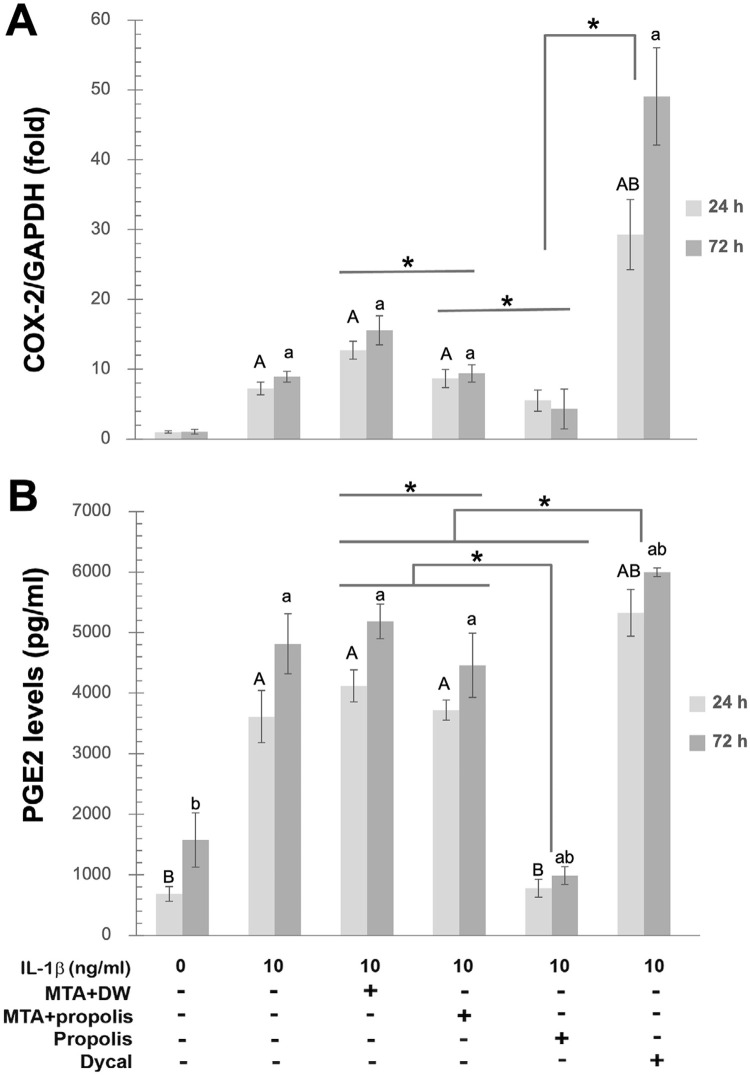
Significantly reduced levels of COX-2 and PGE2 when Thai propolis extract was used as vehicle for MTA preparation. Bar graphs illustrating folds of COX-2 mRNA expression, normalized by GAPDH mRNA expression, in the HDPCs’ lysates of samples in relation to that in the unstimulated HDPCs, set to 1.0 (A) and PGE2 levels in the HDPCs’ conditioned media in pg/ml (B). IL-1β-stimulated HDPCs were treated for 24 h (light gray) or 72 h (dark gray) with 1.5 ml of the eluate released from MTA mixed with distilled water (DW), from MTA mixed with Thai propolis extract at 0.75 mg/ml, or from Dycal®, or Thai propolis extract at 0.75 mg/ml. Error bars = standard deviation; * = *p*<0.05. A = *p*<0.05, compared to unstimulated HDPCs at 24 h; a = *p*<0.05, compared to unstimulated HDPCs at 72 h; B = *p*<0.05, compared to IL-1β-stimulated HDPCs that were not treated for 24 h; b = *p*<0.05, compared to IL-1β-stimulated HDPCs that were not treated for 72 h.

As a gene control for inflammatory responses of HDPCs to different agents tested in this study, we determined COX-2 mRNA expression and PGE2 levels. It was shown that treatment with the eluate of MTA mixed with distilled water or with the propolis extract for 24 or 72 h failed to significantly suppress COX-2 mRNA upregulation or PGE2 elevation upon stimulation of HDPCs with IL-1β at 10 ng/ml, whereas treatment with the propolis extract by itself at both 24 and 72 h was found to significantly diminish the COX-2 upregulation and the PGE2 raise (*p*<0.05; Figure 6A and B, respectively). A significant decrease in COX-2 and PGE2 was noted by treatment with the eluate of MTA mixed with the propolis extract as compared to those by treatment with the eluate of MTA mixed with distilled water (Figure 6A and B). In contrast to significant decreases in MMP-2 expression and activity by treatment with the eluate of Dycal^®^ at both 24 and 72 h (Figure 4 and 5, respectively), significant increases in COX-2 expression and PGE2 levels were instead found (Figure 6A and B, respectively).

## Discussion

To the best of our knowledge, this study is the first to investigate the effects of Thai propolis extract mixed with MTA on MMP-2 activity, COX-2 expression, and PGE2 levels in IL-1β-stimulated HDPCs. However, previous studies have shown an improvement of the biological properties of calcium hydroxide when mixed with propolis and used as a pulp capping material, by producing more type 1 collagen, while diminishing the pro-inflammatory signaling molecule NF-kB in the pulp tissue, when compared to use of calcium hydroxide by itself.^23^ Even though vehicles for MTA preparation other than water have not yet been recommended, this study is at the verge of investigating the pulpal repair mechanisms initially by MMP-2 modulation when MTA was mixed with propolis.

As such, our findings suggest that Thai propolis extract holds promising biological properties to not only dampen COX-2 and PGE2 levels in inflamed HDPCs,^18^ but also to maintain an MMP-2 expression and activity when used as a vehicle for MTA mixing, comparable to that mixed with distilled water, by slightly decreasing MMP-2 mRNA expression and activity, and significantly suppressing COX-2/PGE2 levels. However, while the MMP-2 activity was not significantly reduced by Thai propolis extract mixed with MTA, Thai propolis extract by itself successfully dampened MMP-2 level, suggesting a augmented activation of MMP-2 by MTA and that MMP-2 induction possibly involves multiple signaling pathways. In view of the question as to which active ingredients of Thai propolis extract is responsible for its anti-inflammatory properties, our research group is currently investigating the chemical components of Thai propolis extract. However, earlier reports have elucidated that caffeic acid and polyphenols isolated from propolis inhibited the activities of MMPs in human skin fibroblasts and murine macrophages, respectively.^32,33^ Direct relations between propolis’ active ingredients and the MMPs in dental pulp cells warrant further investigations. It is noteworthy that the MMP-2 activity, but not the MMP-9 activity, was predominantly detected in the conditioned medium of HDPCs as assayed by gelatin zymography, which is consistent with a previous finding.^34^ The setting time of MTA mixed with Thai propolis extract was not different from that of MTA mixed with distilled water at the same ratio of 3:1 (data not shown) with no sediment observed in DMEM, immersed with MTA mixed with the propolis extract for 24 h (Figure 1D). However, it is still necessary to determine whether Thai propolis extract mixed with MTA modulate reparative/tertiary dentinogenesis via MMP-mediated collagen synthesis signaling via further *in vivo* experiments.

Generally, expression and activity of MMP enzymes can be separately controlled at multiple levels, including transcriptional, post-transcriptional, translational, and post-translational levels.^35^Upon stimulation of HDPCs with IL-1β at 10 ng/ml for 24 h, there was no significant upregulation of MMP-2 mRNA expression (Figure 4), whereas the MMP-2 activity was found to be significantly augmented (Figure 5A). As with this opposite finding, treatment of IL-1β-primed HDPCs with the eluate of MTA at 24 or 72 h further induced the expression of MMP-2 mRNA (Figure 4), while it indeed decreased the MMP-2 activity (Figure 5) when compared to stimulation with IL-1β. The discrepancies between the MMP-2 gene expression and the enzymatic activity of MMP-2 upon stimulation with IL-1β or treatment with MTA as previously mentioned may have emphasized the significance of post-transcriptional regulation of MMP-2. Previous laboratory investigations have suggested that post-translational modification plays a significant role in regulating the MMP-2 functions^36^ and that chlorhexidine targeted MMP-2 thereby directly inactivates its enzymatic activity.^37^ The post-translational regulation of MMP-2 is exemplified by inhibition of the MMP-2 activity upon treatment with 0.2% (v/v) of chlorhexidine in dental pulp cells.^32^ Consequently, in this study, treatment of HDPCs with chlorhexidine served as a positive control for inhibition of the MMP-2 activity in the conditioned medium. The proposed mechanisms, by which chlorhexidine suppresses the MMP-2 activity, include a competitive binding with calcium ions that are necessary for the catalytic activity of MMP-2^38^ and/or an inhibitory reaction between chlorhexidine and the sulfhydryl group or the cysteine residue at the active site of MMP-2.^37,39^

The cytotoxicity of MTA appears to be lower than that of Dycal^®^.^26^ The toxicity of the latter was evident by a significant decrease in the percentages of cell viability at the three treatment periods, including 24, 48, and 72 h. At the molecular level, aberrantly decreased MMP-2 expression/activity along with increased COX-2 expression and PGE2 levels were noted for HDPCs treated with the eluate of Dycal^®^. Dampened MMP-2 activity by Dycal^®^ was likely due to the suppression of MMP-2 at alkaline microenvironments.^40^ However, an unchanged MMP-2 phenotype of HDPCs stimulated with the eluate of MTA, regardless of a high pH induction similar to Dycal^®^,^41^ suggests a unique response mechanism of dental pulp to MTA. By contrast, treatment of the dental pulp cells with MTA showed no obvious morphological changes in comparison with the untreated control cells, and even enhanced cell viability following a longer period of incubation with MTA.^26^ Nevertheless, although MTA was not found to be toxic to HDPCs (Figure 2B), it was able to activate inflammation-related molecules by upregulating MMP-2 (Figure 4) and COX-2 (Figure 5) mRNA expressions, which is in line with previous studies.^42,43^

In summary, the highest non-toxic dose of Thai propolis extract at 0.75 mg/ml mixed in MTA can potentially decrease expressions and activities of inflammation-related molecules, including MMP-2 and COX-2 in comparison with those of MTA mixed with distilled water in IL-1β-stimulated HDPCs. The level of MMP-2 was maintained by Thai propolis extract used as vehicle, although a slight inhibition was observed. Thai propolis extract mixed in MTA is likely to be clinically beneficial for vital pulp therapy as a pulp capping material in order to simultaneously alleviate inflammation and promote mineralization. Nonetheless, due to the nature of *in vitro* experiments, proper titration of Thai propolis extract concentration that yields for optimal pulpal immune response is still needed. Therefore, further *in vivo* studies are required for elucidating an orchestration of various intracellular signals in HDPCs in the protection mechanisms against pulpal inflammation and tertiary dentine synthesis as modulated by MTA mixed with Thai propolis extract. Furthermore, physical properties of MTA mixed with Thai propolis extract need to be examined, as well as alterations in expressions of membrane type 1 matrix metalloproteinase and of tissue inhibitor of MMP-2 together with their interactions with the MMP-2 activity and collagen metabolism in cultured HDPCs treated with different pulp capping materials.
